# 3D-printed total humeral prosthesis with shoulder preservation in the treatment of humeral osteosarcoma: a case report

**DOI:** 10.3389/fonc.2026.1764651

**Published:** 2026-05-14

**Authors:** Haocheng Cui, Kai Zheng, Ming Xu, Qian Chen, Kai Zhai, Wenqiang Xing, Xiuchun Yu

**Affiliations:** Orthopedic Department, 960 Hospital of People’s Liberation Army, Jinan, Shandong, China

**Keywords:** 3D printing, bone neoplasms, joint-preservation, shoulder function, total humeral replacement

## Abstract

**Background:**

For large malignant tumors of the humerus involving extensive portions of the diaphysis, total humeral replacement represents a critical option for limb salvage and functional reconstruction. However, maximizing preservation of shoulder and elbow joint function remains a significant challenge for surgeons.

**Case presentation:**

We report a case of a 24-year-old male with chondroblastic osteosarcoma of the humerus. Preoperative biopsy confirmed a high-grade sarcoma consistent with osteosarcoma, most likely the chondroblastic subtype. He received two cycles of neoadjuvant chemotherapy comprising cisplatin, adriamycin, and ifosfamide, along with the PD-1 inhibitor sintilimab. Subsequently, right humeral tumor resection was performed, followed by reconstruction with a 3D-printed prosthesis that preserved the shoulder joint and incorporated hemi-elbow arthroplasty. Postoperatively, an elbow brace was worn for four weeks, with the elbow maintained at 15° of flexion. After suture removal, chemotherapy and PD-1 inhibitor therapy were resumed. At the 9-month follow-up, the patient maintained functional use of the limb and performed activities of daily living independently. Physical examination revealed good shoulder and elbow motor function. Imaging confirmed proper prosthesis placement and robust osseointegration. Artifact-free CT scans of the shoulder and chest showed no evidence of local recurrence or distant metastasis.

**Conclusion:**

The semi-elbow prosthesis—designed using 3D printing technology while preserving the patient’s humeral head—effectively maximizes preservation of shoulder and elbow joint function and enhances treatment satisfaction in young patients.

## Introduction

The humerus is a common site for primary bone tumors and is also one of the frequent locations for bone metastases (1). With advancements in neoadjuvant chemotherapy, surgical techniques, medical imaging, and implant manufacturing technologies, limb-salvage surgery has become the standard of care in current clinical practice (2, 3). However, a large malignant tumor of the humerus may involve an extensive portion of the diaphysis and may present with skip metastases at either the proximal or distal end, necessitating wide resection. This often results in insufficient residual host bone to permit reliable allograft transplantation or stable fixation of a prosthetic stem (4, 5). In this context, total humeral replacement (THR) is considered a safe and viable option for reconstruction (6).

Compared with other treatment modalities, THR offers several advantages, including greater patient emotional acceptance, preservation of functional capabilities in the elbow and hand, and no interference with the efficacy of tumor resection. Furthermore, this procedure provides immediate upper limb stability and eliminates concerns regarding nonunion or delayed union—benefits that are particularly valuable when adjuvant radiotherapy or chemotherapy must be initiated promptly after surgery (4, 7). However, THR is associated with certain inherent limitations, and the most frequently reported complication is shoulder joint dysfunction (8, 9). Most patients following THR are unable to achieve active shoulder abduction against gravity, primarily due to intraoperative resection of rotator cuff tendons and the deltoid muscle (10, 11). The extensive removal of rotator cuff musculature and the humeral head during surgery is a key factor contributing to proximal migration of the prosthetic humeral component in most cases. Furthermore, some patients may experience postoperative pain secondary to acromial impingement (12). In addition, the elbow joint component of the total humeral prosthesis features a hinged design that requires fixation of the intramedullary stem within the medullary canal of the ulna. Although this configuration enhances elbow joint functionality, it may lead to substantial stress concentration at the liner and in the periprosthetic region surrounding the intramedullary stem during flexion and extension, potentially resulting in prosthetic wear and loosening over time (4, 13, 14).

Given the aforementioned limitations of THR, it is imperative to investigate a reliable reconstructive approach that maximizes preservation of shoulder and elbow joint function in patients. Owing to ongoing advancements in 3D printing technology and growing research into limb-salvage surgery guided by the principle of joint preservation, this study reports a case in which a novel humeral prosthesis was designed and fabricated using 3D printing techniques. This prosthesis preserves the patient’s native humeral head at the proximal end and is secured to it via a short intramedullary stem combined with a lateral locking plate. The proximal prosthesis–bone interface is fabricated with porous titanium structures using selective laser melting (a 3D printing technique) to promote effective osseointegration and ensure long-term implant stability. At the distal end, a customized metallic segment is additively manufactured based on preoperative computed tomography (CT) data of the patient’s humerus to precisely match the articular surfaces of the ulnar olecranon and radial capitellum, thereby enabling anatomical conformity and biomimetic reconstruction. This article details the design principles, clinical presentation, and postoperative outcomes of the prosthesis, while critically evaluating its advantages and limitations. The study aims to improve patient outcomes by systematically investigating and summarizing the clinical application of humeral prostheses that preserve the native humeral head—thereby providing clinicians with more comprehensive and actionable insights.

## Case presentation

### Clinical data

#### Diagnosis and tumor therapy

**Figure 1:**
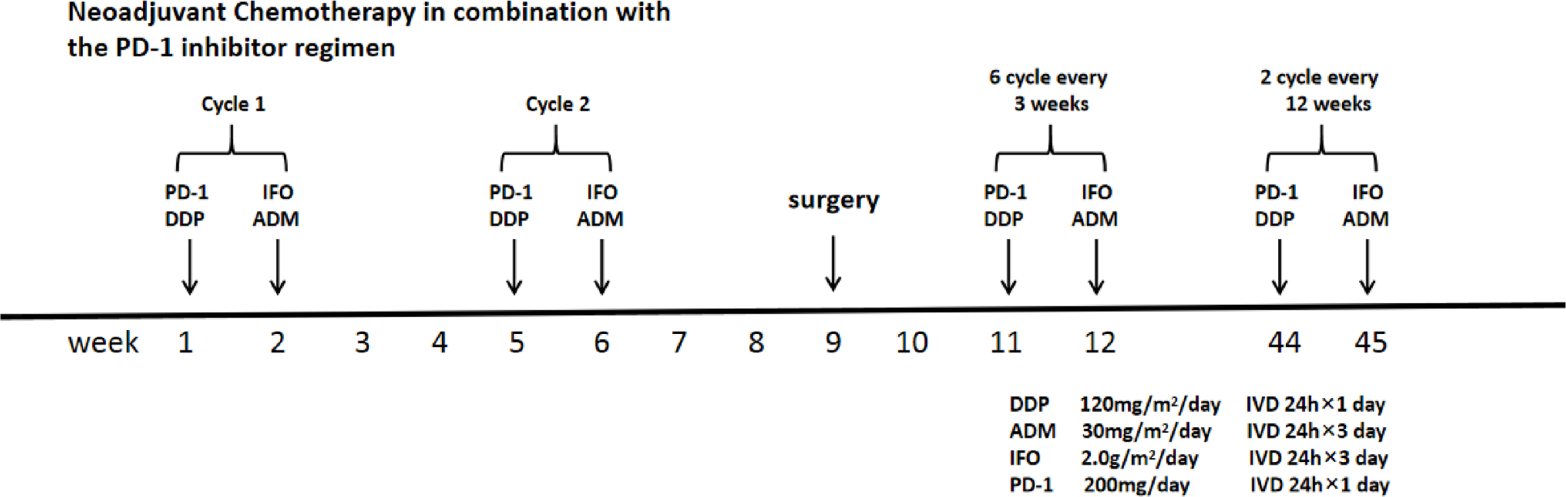
Neoadjuvant chemotherapy in combination with the PD-1 inhibitor regimen. DDP, cisplatin; IFO, ifosfamide; ADM, adriamycin; PD-1 inhibitor, sintilimab.

A 24-year-old male presented to a local hospital with persistent pain and discomfort in his right upper arm for two weeks. Biopsy confirmed a diagnosis of high-grade sarcoma, suspected to be chondroblastic osteosarcoma. The patient underwent one cycle of preoperative neoadjuvant chemotherapy with cisplatin and adriamycin. However, the chemotherapy was ineffective: swelling and pain did not improve, and imaging and clinical findings indicated tumor progression. Subsequently, the patient presented at our hospital. A physical examination conducted upon admission revealed swelling of the right upper arm, with the proximal circumference of the elbow joint measuring 34 cm—7 cm greater than that of the contralateral side. Tenderness was present in the middle and distal thirds of the right upper arm, and skin temperature was elevated. Skin color remained normal. A firm, non-tender mass measuring approximately 15 × 8 cm was palpable in the right upper arm. Range of motion of the right elbow and shoulder joints was within normal limits, and the Visual Analog Scale (VAS) score was 5. Upon admission, the patient received two cycles of DIA-regimen chemotherapy (120mg/m^2^/day cisplatin, 2.0 g/m^2^/day ifosfamide, and 30mg/m^2^/day adriamycin) and two doses of the PD-1 inhibitor (200mg/day sintilimab) (**Figure 1**).

**Figure 2:**
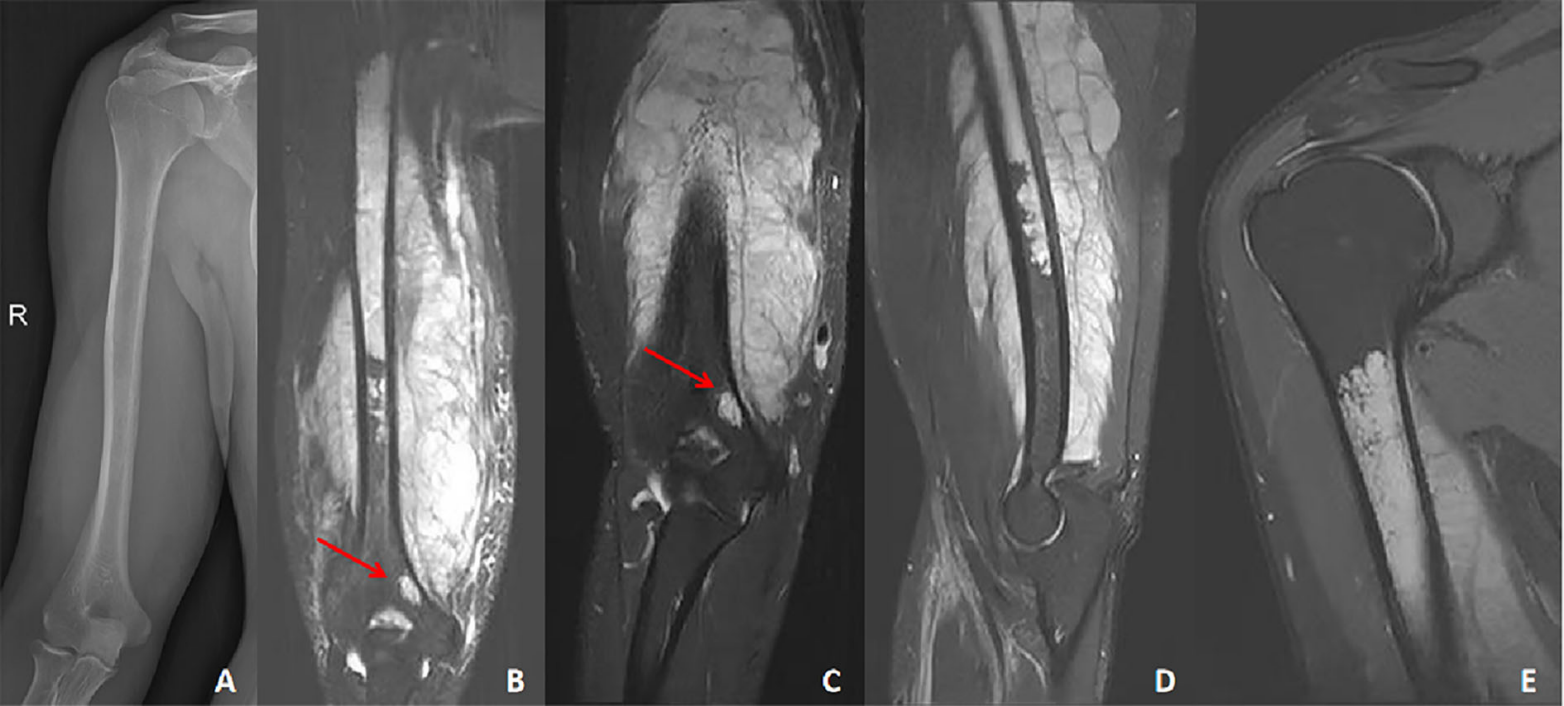
A 24-year-old patient with chondroblastic osteosarcoma of the humerus. **(A)** Pre-treatment radiography revealed normal bone mineralization of the humerus, with no evidence of osseous abnormality; however, a soft-tissue mass was observed in the mid- and distal shaft. **(B)** Pre-treatment MRI demonstrated a large tumor occupying the medullary cavity of the humeral shaft and extending into the surrounding soft tissues. A skip lesion was also identified at the humeral condyle (red arrow). **(C, D)** Post-treatment MRI showed marked reduction in tumor volume with well-defined margins, resolution of peritumoral edema, and persistence of a skip metastasis at the distal humeral condyle (red arrow). **(E)** Post-treatment MRI confirmed no tumor involvement of the humeral head.

Following the combined treatment, swelling and pain in the patient’s right upper arm were notably alleviated. Preoperative physical examination revealed that the circumference of the proximal aspect of the right elbow joint was 33 cm—6 cm greater than that of the contralateral side. The mass in the right upper arm measured approximately 10 × 6 cm. Range of motion at the right elbow and shoulder joints was normal, and the VAS score was 3. Repeat radiographic evaluation of the right humerus revealed normal bone density and a well-defined soft-tissue mass surrounding the humeral shaft. Magnetic resonance imaging (MRI) of the right humerus demonstrated a stable tumor volume and well-defined margins. A large soft-tissue mass occupying both the medullary cavity and the surrounding soft tissues of the humeral shaft was observed; however, the humeral head remained uninvolved. Additionally, a skip metastasis was identified at the distal humeral condyle (**Figure 2**). Preoperative PET-CT examination revealed increased metabolic activity in the right humeral shaft and surrounding soft tissues, with no evidence of distant metastasis. The patient’s Enneking stage is III-B.

#### Design and 3D customized humeral prosthesis

**Figure 3:**
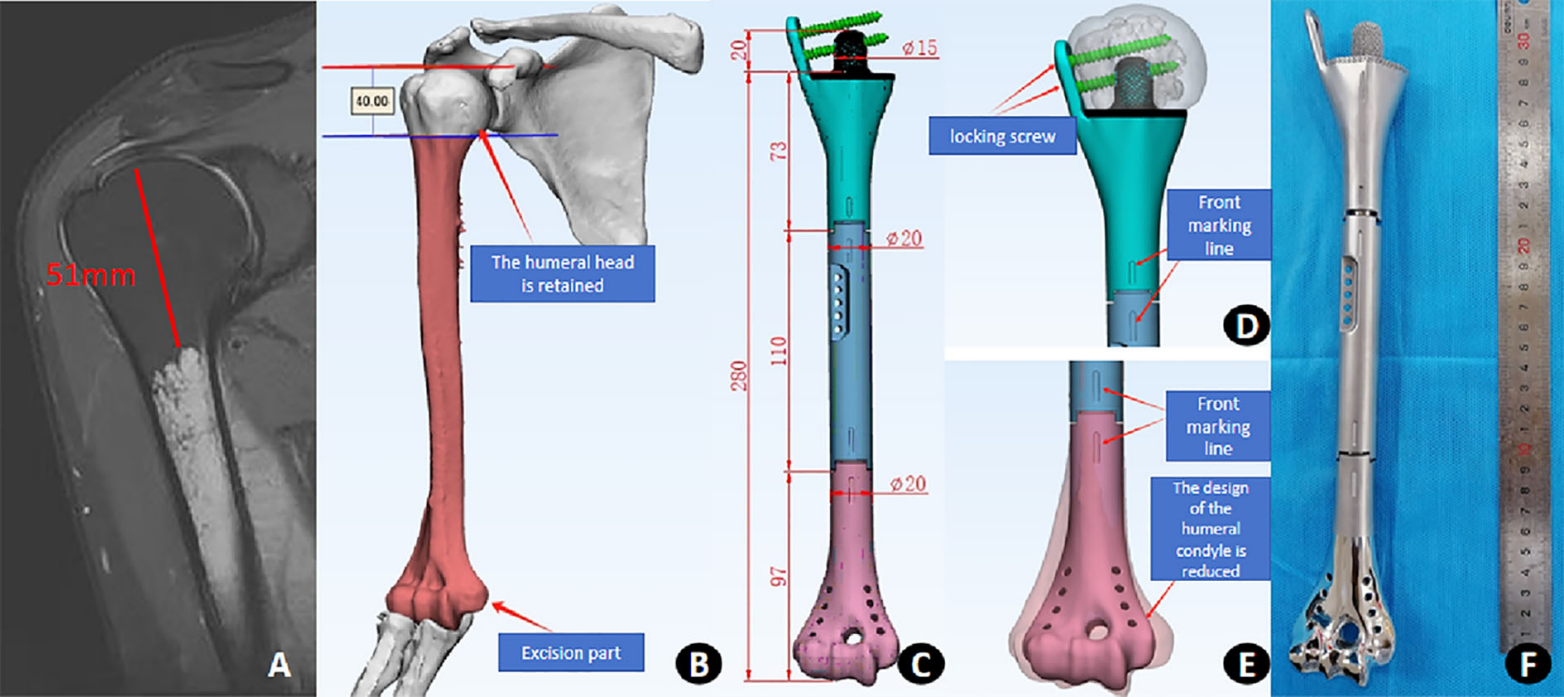
Preoperative imaging and prosthesis design. **(A)** Preoperative MRI demonstrated that the proximal 5 cm of the humerus was free of tumor invasion. **(B)** After CT scanning, the patient’s right humerus was three-dimensionally reconstructed. During surgery, a 4-cm margin of healthy bone at the proximal humerus will be preserved. The red-marked area indicates the pathological tissue to be resected. **(C)** The semi-elbow joint humeral prosthesis is designed in three structural segments: proximal, middle, and distal. **(D)** The proximal 4 cm of the humeral head is preserved, and the prosthesis is fixed using a lateral plate and a short intramedullary stem. Alignment marks on each segment ensure correct orientation and prevent rotational misalignment during implantation. **(E)** The distal component of the prosthesis is designed to be 15% smaller than the native humeral condyle and incorporates dedicated channels for fixation of surrounding soft tissues. **(F)** Personalized prosthetic devices.

The patient is a young male with a strong preference for limb preservation. Combined immunotherapy and chemotherapy have yielded favorable therapeutic outcomes. A personalized semi-shoulder joint humeral prosthesis was designed to preserve the patient’s native humeral head, which is well suited to his clinical and functional requirements. Following two cycles of neoadjuvant chemotherapy and immunotherapy prior to surgery, thin-section CT scans (1.5 mm slice thickness) were performed on the patient’s right humerus to acquire imaging data. MRI findings indicated that the proximal 5 cm of the humeral head remained free of tumor invasion. Based on the aforementioned examination results and data, the prosthesis was designed as follows: The proximal osteotomy plane was set at the proximal 4 cm of the humerus. The proximal prosthetic component was secured to the residual humeral segment using a 2-cm intramedullary short stem in conjunction with a lateral fixation plate. Both the intramedullary stem and the prosthetic-bone interface underwent porous surface treatment to enhance osseointegration. The humeral shaft portion of the prosthesis is 110 mm in length. The distal humeral condylar component is designed to be 15% smaller than the native humeral condyle, with eight precisely positioned holes provided for soft tissue and ligament fixation. The surface of the distal humeral condyle prosthesis is polished to ensure smooth articulation. The three-part prosthesis system is assembled using an axially impacted cylindrical stem and a Morse taper connection. Vertical alignment markings are placed on the anterior aspect of each connecting segment to facilitate accurate intraoperative assembly and prevent rotational misalignment (**Figure 3**). The prosthesis was manufactured using electron beam melting (EBM) technology. The humeral shaft component was produced from a cobalt–chromium–molybdenum alloy via casting, whereas the proximal and distal components were fabricated from TC4 (Ti-6Al-4V) forged titanium alloy. These devices were designed and processed in collaboration with Shandong Weigao Orthopedic Device Co., Ltd. (China).

#### Surgical procedure

The patient was positioned supine with secure fixation of the head and endotracheal tube. This surgical procedure utilizes an extended humeral approach that integrates the standard deltoid-pectoral approach to the shoulder, the anterolateral approach to the humerus, and the posterior approach to the elbow joint (15, 16).

**Figure 4:**
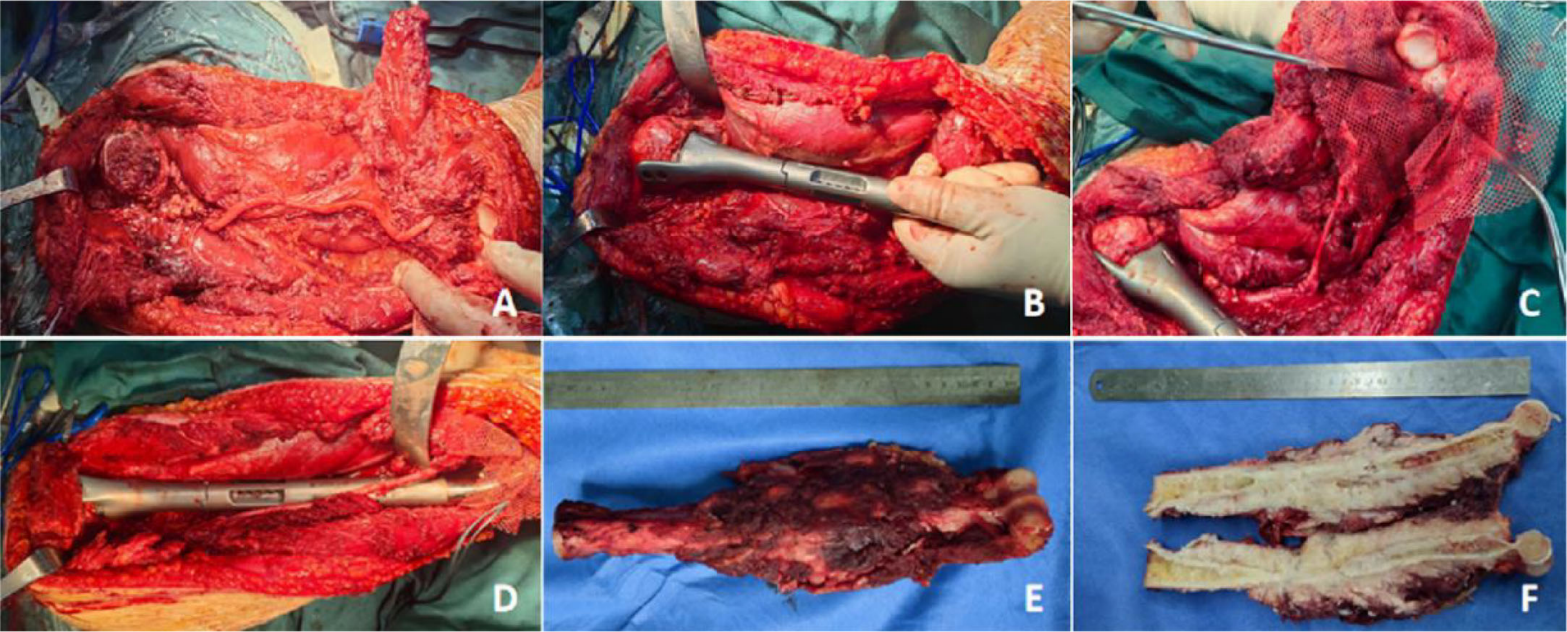
Surgical procedure and tumor specimen. **(A)** The tumor segment was completely resected while preserving the humeral head and protecting the neurovascular bundle. **(B)** The proximal humeral prosthesis was implanted, secured to the humeral head, and connected to the shaft prosthesis. **(C)** The elbow joint capsule was reconstructed around the radial head and the ulnar olecranon using mesh. **(D)** The elbow joint was flexed, the joint capsule was securely sutured, and tendons and adjacent soft tissues near the prosthesis were reconstructed and repaired. **(E, F)** The en bloc resected tumor specimen—including its cross-sectional view—confirms a safe proximal osteotomy margin.

The cephalic vein is exposed and preserved. The anterior and lateral portions of the deltoid muscle are partially divided, and the long head of the biceps tendon is released (not sectioned) to expose the proximal humerus. A point 4 cm distal to the apex of the humeral head-and the intertubercular groove-are marked for anatomical reference. The distal end was further dissected, and the soft-tissue mass—with a margin of adjacent normal muscle—was excised en bloc. The radial nerve was identified and exposed along the mid-to-distal humerus. The distal posterior aspect of the elbow joint was exposed. The ulnar nerve groove was incised, and the nerve was carefully dissected, mobilized proximally, and protected. The proximal humerus was osteotomized using an ultrasonic osteotome, and bone samples were harvested from the medullary cavity of the residual segment for histopathological analysis to confirm the absence of tumor involvement. After transection, the humerus was exposed from proximal to distal. The tumor’s nutrient vessels were ligated, the median nerve was dissected and protected, the elbow joint capsule was incised, and the tumor segment was fully resected. First, the proximal prosthesis was implanted by preparing the medullary canal of the residual humeral head and securely positioning the component. Align the intertubercular groove with the anterior reference line on the prosthesis, then insert two locking screws through the lateral plate to achieve rigid fixation. Subsequently, reattach the proximal stumps of the deltoid muscle and the long head of the biceps brachii using non-absorbable sutures. Next, assemble the humeral shaft with the distal humeral prosthesis, ensuring precise alignment of the anterior reference marks during coupling. Reconstruct the joint capsule at the ulnar olecranon and radial head using synthetic mesh. Reduce the elbow joint and securely suture the ligamentous structures and capsular reinforcements. Following reconstruction, evaluate passive range of motion and assess joint stability bilaterally–specifically across the medial and lateral aspects (**Figure 4**).

#### Postoperative treatment

Antibiotics were administered both preoperatively and postoperatively as a prophylactic measure against infection. A multimodal analgesia regimen was implemented for pain management. The drainage tube was removed when the daily postoperative negative-pressure drainage volume decreased to less than 30 mL. Sutures were removed two weeks after surgery. An elbow brace was worn for four weeks postoperatively to maintain the elbow at 15°of flexion, while patients were encouraged to mobilize their shoulder and hand joints. The postoperative pathology confirmed chondroblastic osteosarcoma, with a tumor necrosis rate of 70% and a HUVOS grade of II. After suture removal, the DIA regimen chemotherapy was resumed in combination with PD-1 inhibitor therapy. The chemotherapy combined with immunotherapy regimen is shown in **Figure 1**.

#### Outcome and follow-up

**Figure 5:**
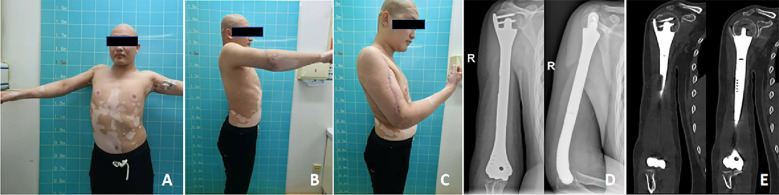
Nine months postoperatively, the patient’s shoulder and elbow joint function was assessed using imaging. **(A–C)** The patient’s right shoulder joint demonstrated active abduction to 75°, forward flexion to 80°, while the elbow joint exhibited a passive range of motion from 0°to 90°. **(D)** Anteroposterior and lateral radiographs of the right humerus showed proper prosthesis positioning, well-integrated bone-prosthesis interfaces, and good elbow alignment. **(E)** An artifact-free CT scan of the right humerus revealed successful integration at the 3D-printed prosthesis-bone interface, with appropriate alignment of the right elbow joint.

The patient received six monthly cycles of the postoperative DIA regimen chemotherapy combined with PD-1 inhibitor therapy. In the ninth month after surgery, he received his seventh cycle of treatment. Imaging evaluations, including X-ray and CT scans of the affected limb, were performed at each admission; no signs of local recurrence or disease progression were observed. At nine months postoperatively, shoulder and elbow joint function was clinically assessed, and imaging was repeated. The patient subjectively reported that function of the operated limb was largely preserved, with the ability to perform essential activities of daily living. Physical examination revealed symmetrical alignment of the bilateral shoulder joints, with no significant contour alteration compared to the contralateral side. The right shoulder joint demonstrated a satisfactory range of motion: abduction to 75°, forward flexion to 80°, extension to 30°, and full elevation without restriction (**Figure 5**). Muscle strength in all directions around the right shoulder was graded as Grade IV on the Medical Research Council (MRC) scale. Functional assessment yielded a right shoulder ASES (American Shoulder and Elbow Surgeons) score of 84 points (17) and an MSTS (Musculoskeletal Tumor Society) score of 25 points (18). The range of the right elbow was recorded as 0°to 90°, with a Mayo Elbow Performance Score (MEPS) of 80 points (19).

On the X-ray, no radiolucent line is observed at the interface between the prosthesis and the bone; no periprosthetic osteoporosis is evident; and the prosthesis remains in stable position. Using the original artifact-free CT data, we measured a bone volume fraction (BV/TV) of 25% and a bone–implant contact (BIC) rate of 60%. These findings indicate satisfactory osseointegration 9 months postoperatively (**Figure 5**).

## Discussion

### Functional issues and causes of limb dysfunction after THR

THR represents one of the limited reconstructive options available in cases of extensive humeral involvement or skip lesions. However, impaired shoulder joint function remains a primary concern associated with this procedure (4, 9, 20–22). Li et al. (9) reviewed 34 patients after total humeral replacement (THR). At final follow-up, 20 patients were alive, with a mean ASES score of 79 (range: 63-93). The main failure mechanisms were tumor recurrence and soft tissue complications. Upper limb function was satisfactory, with preserved hand and elbow function, though shoulder function remained limited. Natarajan et al. (4) reviewed 11 patients who received THR for malignant tumors. Postoperatively, all patients had better passive motion than active movement, with no one able to actively raise their arm above shoulder level. While some had limited elbow extension, hand function remained intact in all. Imaging showed prosthetic head instability or proximal migration in every case. Gonzalez et al. (8) also noted in their most recent systematic review that approximately one-third of all humeral arthroplasties fail, primarily due to tumor progression and prosthetic infection. However, among surviving cases, hand and elbow functions are generally well preserved, whereas shoulder joint function remains significantly impaired. **Table 1** summarizes limb function outcomes after THR reported in the literature.

**Table 1:** Clinical report on the use of total humeral replacement for malignant tumor of the humerus.

Author	Year	Number of cases	Mean follow-up(month)	Outcome	Function	Shoulder function	Elbow and hand function
Puri et al (20).	2012	19	41	5 local recurrences,11 patients alive; The implant survival was 95%	MSTS 73%	All patients had limited shoulder function	Fair elbow, good hand.
Natarajan et al (4).	2011	11	66	2 patients died due to metastasis. 1 patient amputation for local Recurrence;	MSTS 80%.	All patients had limited shoulder function	Fair elbow, good hand.
Wafa et al (21).	2015	34	120	10-year implant survival rate was 90%;4 developed periprosthetic infection, 1 had postoperative radial nerve palsy, 3 had proximal migration of the prosthesis, 3 required elbow joint liner replacement	MSTS 83%.	All patients had limited shoulder function. None patients was able to actively elevate the shoulder to more than 60° in the sagittal, coronal, or scapular plane	12 patients had an average flexion deformity of the elbow of 15°(range, 10°– 30°) but only 3 patients had active elbow flexion of less than 100°.
Schneider et al (22).	2021	31	42	18 patients died of disease, 3 were living overseas and 1 was untraceable. At the last follow-up, 9 patients with functional outcome data.	MSTS 87%, ASES 83	No pain in shoulder; The active or weight-bearing movement of the shoulder joint is limited	Good hand
Kotwal et al (6).	2016	20	42.9	11 patients alive, 9 patients died of metastatic disease. The survival rate of the implants at 5 years was 87.1% and at 10 years was 65.3%	MSTS 71.5%	All patients had limited shoulder function	Fair elbow, good hand.
Li et al (9).	2025	34	78.5 ± 72.6	20 patients alive;The 5-year, 10-year and 15-year overall survival rates of the prosthesis were 94% respectively.	MSTS 78%; ASES 79%	All patients had limited shoulder function	Fair elbow, good hand.

MSTS, Musculoskeletal Tumor Society.

ASES, American Shoulder and Elbow Surgeons.

Joint stability is fundamental to proper joint function. The shoulder joint is widely regarded as the most inherently unstable joint in the human body, primarily due to the marked disparity in size between the humeral head and the glenoid fossa. Its stability is maintained through the contributions of the glenoid labrum-which deepens the glenoid cavity to improve articulation-and the rotator cuff: a functional complex of peri-scapular soft tissues that collectively provide dynamic stabilization to the joint. When metaphyseal bone defects result in significantly shortened residual bone segments, hemiarthroplasty or total joint replacement often necessitates resection of the humeral head. Following implantation of a proximal humeral prosthesis, the prosthetic humeral head and the native glenoid cavity exhibit suboptimal congruence, compromising joint stability relying solely on surface articulation. Moreover, biological integration between soft tissues and the prosthesis does not occur, resulting in reduced structural integrity compared with the native tendon-to-bone interface (23).

Compared with prostheses used in other anatomical regions, proximal humeral prostheses exhibit the highest survival rate; however, postoperative functional outcomes remain relatively poor. This is primarily attributed to extensive resection of muscles and ligaments during joint replacement surgery, as well as the residual joint capsule’s insufficient capacity to adequately envelop and stabilize the metallic humeral head-leading to a high incidence of prosthetic dislocation (24).

When the patient’s native humeral head can be preserved while ensuring complete tumor resection, shoulder joint function and stability are maximized. Humeral head-sparing prostheses preserve the anatomical integrity of the humeral head, thereby maintaining natural shoulder biomechanics; they also provide superior initial stability and faster functional recovery compared with conventional joint replacements.

### Limb salvage surgery with joint preservation

Limb salvage surgery for malignant bone tumors is typically classified according to the reconstruction method employed-such as prosthetic implantation or massive allogeneic bone grafting following tumor resection. In the literature, limb salvage surgery is further classified based on joint integrity preservation into two main types: joint-preserving limb salvage (JPLS), which maintains the native joint structure, and joint-replacing limb salvage (JRLS), which involves resection of the affected joint and/or its replacement with a prosthesis. In recent years, advances in imaging technology have enabled more accurate preoperative assessment of tumor invasion extent. Several scholars have emphasized the importance of precisely evaluating both the location and invasive range of the tumor prior to surgery, while striving to preserve the integrity of the patient’s native joint structures to optimize postoperative limb function (25).

The primary objective of JPLS is to preserve joint structural integrity to the greatest extent possible while strictly adhering to oncological resection principles, avoiding any increase in the risk of life-threatening complications, and ultimately improving postoperative limb function to enhance patient satisfaction with limb-sparing surgery (25).

The indications for JPLS (26–28) are as follows: (1) The tumor should be located in the diaphysis or metaphysis of the bone; in pediatric patients, this criterion applies only when the growth plate remains open. (2) Prior to surgery, the extent of tumor invasion must be accurately assessed. Joint structure should be preserved as much as possible while ensuring complete tumor resection. Preoperative MRI is recommended due to its high accuracy in delineating tumor margins, and histopathological examination after surgery is required for confirmation. (3) For chemotherapy-sensitive tumors, neoadjuvant chemotherapy should be administered before surgery, following an effective preoperative treatment regimen.

Compared with JRLS, JPLS imposes higher demands on the surgeon, including meticulous preoperative planning of the tumor resection extent and reconstructive strategy, precise intraoperative determination of the osteotomy boundaries, postoperative histopathological confirmation of negative surgical margins, and comprehensive management of postoperative complications and functional limb rehabilitation.

### The application of 3D printing technology in prostheses with joint preservation

For joint preservation, reconstructive options for proximal humeral defects following extensive tumor resection include autologous bone grafting, allogeneic bone grafting, and prosthetic reconstruction (29–31). Autologous fibular grafts exhibit favorable biocompatibility and osteoconductive properties. However, after resection, the anatomical mismatch between the fibular head and the proximal humerus may compromise interfacial bone integration, potentially leading to complications such as bone resorption and fracture (32, 33). Allogeneic bone grafts of suitable size and shape can achieve optimal interface integration between the graft and host bone; however, immune-mediated rejection and impaired bone healing remain significant clinical challenges (34). Furthermore, prolonged immobilization is typically required following extensive allogeneic bone transplantation, inactivated reimplantation with internal fixation, or autologous fibular grafting–all of which are associated with a high incidence of nonunion. Additionally, inflammatory wound exudate resulting from host immune responses to allogeneic tissue may delay the initiation of radiotherapy and chemotherapy, thereby compromising overall treatment efficacy. Tumor prosthesis replacement surgery is characterized by its technical simplicity and short operative duration; patients can begin early postoperative functional rehabilitation, and the procedure is associated with a low incidence of wound-related complications. Moreover, it does not interfere with subsequent radiotherapy or chemotherapy, making it a widely adopted treatment option among clinicians today.

Tumor-specific artificial joint prostheses have demonstrated satisfactory reconstructive outcomes at certain anatomical sites; however, functional reconstruction remains suboptimal in the shoulder, elbow, and ankle joints; midshaft bone defects of long bones; and pelvic regions. The underlying reasons are as follows: First, traditional forging techniques cannot achieve precise geometric conformity between the prosthesis and the bone defect, nor can they fabricate prostheses with a gradient elastic modulus–both of which are essential for minimizing stress shielding and optimizing mechanical load transfer. Second, current interface coating (e.g., plasma-sprayed) technologies have not yet achieved clinically satisfactory levels of osseointegration. Furthermore, bone defects following tumor resection exhibit substantial interpatient variability, and conventional subtractive manufacturing methods are inadequate for accurately fulfilling such patient-specific anatomical requirements.

3D printing technology has enabled a novel approach to the personalized fabrication of titanium alloy implants for hard tissue replacement. By precisely engineering porous metallic trabecular structures at the prosthesis-bone interface, this technology facilitates optimal osseointegration, thereby enhancing the long-term stability and performance of artificial prostheses. Guo et al. (35) proposed the PAMFOS principle for reconstructing bone and joint defects through the application of 3D printing technology in prosthesis fabrication: P–precision, A–articulation, M–material, F–fixation, O–osseointegration, S–soft tissue.

In the management of this patient, we adhered to the aforementioned principles. Building upon effective pharmacological therapy, a customized prosthesis was designed based on detailed preoperative planning to meet the patient’s individualized requirements. The native humeral head was preserved at the proximal end, thereby maximizing shoulder joint function and stability. At the distal end, we avoided the hinged design used in most total humeral prostheses, as the elbow’s complex motion cannot be replicated by a simple hinge. Moreover, hinged designs may generate high stress at the hinge and stem during activity, increasing wear and the risk of implant loosening (36). A 3D-printed distal humeral hemi-elbow prosthesis was employed to reduce the risk of long-term loosening, providing physiological joint surface conformity while meeting both short-term stability and mobility requirements (37). The semi-constrained elbow joint prosthesis design reduces operative time and simplifies implantation in clinical practice.

### Clinical applications of chemotherapy combined with immunotherapy

Current research on the combination of immunotherapy and chemotherapy has expanded to soft tissue sarcoma, but studies in osteosarcoma remain very limited. Existing evidence suggests that combining conventional chemotherapy with PD-1/PD-L1 pathway inhibitors can modulate the tumor microenvironment, reverse chemotherapy-induced immunosuppression, and thereby enhance antitumor efficacy (38). However, immunotherapy for osteosarcoma is currently limited to a few clinical trials in patients with refractory or advanced disease, and only a small number of patients have benefited from it (39).

At our center, for patients with refractory osteosarcoma who present with distant metastasis at diagnosis or develop local recurrence or distant metastasis after surgery, we attempt treatment with a combination of chemotherapy and immunotherapy. In some patients, median progression-free survival (PFS) has been prolonged. For patients with high-grade osteosarcoma confirmed by biopsy at diagnosis, further immunohistochemical analysis is performed. If PD-L1 expression is positive, adjuvant treatment combining immunotherapy and chemotherapy may be considered.

Based on the above viewpoints, we attempted to treat this chemotherapy-insensitive patient with a combination of immunotherapy and chemotherapy. Currently, no established guidelines or standardized treatment protocols exist to guide such therapy. Sintilimab—the IgG4 humanized anti-PD-1 monoclonal antibody used in this case—has been approved in China for the treatment of classical Hodgkin lymphoma and non-small cell lung cancer (NSCLC) (40, 41).

The regimen of sintilimab combined with chemotherapy is as follows: For patients weighing less than 60 kg, the intravenous infusion dose of sintilimab is 3 mg/kg; for those weighing ≥60 kg, the dose is 200 mg. Immunotherapy is administered throughout the entire treatment course—specifically, two preoperative doses (200 mg each, intravenously, every three weeks), followed by continued administration concurrently with postoperative chemotherapy. Sintilimab is infused intravenously first; cisplatin is initiated 5 minutes after completion of the sintilimab infusion. A total of eight postoperative chemotherapy cycles are planned: the first six cycles are administered every three weeks, and the final two cycles are spaced three months apart. Sintilimab is co-administered with each chemotherapy cycle and is always infused intravenously prior to cisplatin. This patient has now completed the seventh postoperative chemotherapy cycle combined with immunotherapy. Throughout the treatment period, no immune-related adverse events attributable to PD-1 inhibition were observed. Only bone marrow suppression and gastrointestinal adverse reactions occurred, both managed symptomatically.

The main limitations of our study are the relatively short observation period and the lack of testing for biomarkers—such as tumor mutational burden (TMB) and microsatellite instability (MSI)—to predict response to combination immunotherapy.

## Conclusion

3D printing enables the fabrication of customized prostheses that precisely match individual patient anatomy. Preserving the humeral head supports shoulder range of motion and joint stability. The distal humeral prosthesis features a physiologically conforming articular surface. Together, these advantages maximize joint function and improve treatment satisfaction–particularly in younger patients. Moreover, 3D printing provides a practical approach to limb salvage surgery by preserving patients’ native joints.

## Data Availability

The original contributions presented in the study are included in the article/supplementary material. Further inquiries can be directed to the corresponding author.
